# Depatux-M and temozolomide in advanced high-grade glioma

**DOI:** 10.1093/noajnl/vdaa063

**Published:** 2020-06-06

**Authors:** Sophie Hirsch, Cristiana Roggia, Saskia Biskup, Benjamin Bender, Irina Gepfner-Tuma, Franziska Eckert, Daniel Zips, Nisar P Malek, Helmut Wilhelm, Mirjam Renovanz, Ghazaleh Tabatabai

**Affiliations:** 1 Department of Neurology and Interdisciplinary Neuro-Oncology, University Hospital Tübingen, Eberhard Karls University Tübingen, Tübingen, Germany; 2 Hertie Institute for Clinical Brain Research, Eberhard Karls University Tübingen, Tübingen, Germany; 3 Center for Neuro-Oncology, Comprehensive Cancer Center Tübingen-Stuttgart, University Hospital Tübingen, Eberhard Karls University Tübingen, Tübingen, Germany; 4 Center for Personalized Medicine, University Hospital Tübingen, Eberhard Karls University Tübingen, Tübingen, Germany; 5 Department of Genetics, University Hospital Tübingen, Eberhard Karls University Tübingen, Tübingen, Germany; 6 CeGaT and Praxis for Human Genetics, Tübingen, Germany; 7 Department of Diagnostic and Interventional Neuroradiology, University Hospital Tübingen, Eberhard Karls University Tübingen, Tübingen, Germany; 8 Department of Radiation Oncology, University Hospital Tübingen, Eberhard Karls University Tübingen, Tübingen, Germany; 9 Department of Internal Medicine I, University Hospital Tübingen, Eberhard Karls University Tübingen, Tübingen, Germany; 10 Department of Ophtalmology, Division of Neuro-ophtalmology, University Hospital Tübingen, Eberhard Karls University Tübingen, Tübingen, Germany; 11 Department of Neurosurgery, University Hospital Tübingen, Eberhard Karls University Tübingen, Tübingen, Germany

The treatment for multiple progressing high-grade gliomas remains challenging. Amplifications of the epidermal growth factor receptor (EGFR) are frequent events in high-grade glioma. Targeting strategies involving amplified EGFR are therefore considered promising. We present a single-center cohort of 9 advanced EGFR-amplified high-grade gliomas treated with the antibody-drug conjugate (ADC), Depatuxizumab Mafodotin (Depatux-M, ABT-414). Depatux-M acts by an EGFR-antibody-guided delivery of monomethyl auristatin F to molecular-defined target cells. The antitumoral activity will only occur in EGFR-amplified cells but not in the cells of normal tissue without EGFR amplification.^1^ The main adverse event in our cohort was corneal epitheliopathy.

The results of the INTELLANCE 2/EORTC 1410 phase II study of Depatux-M alone and with temozolomide versus temozolomide or lomustine in progressive EGFR-amplified glioblastoma have been recently published.^1^ This trial enrolled patients with the first progression of glioblastoma.

**Figure 1.**
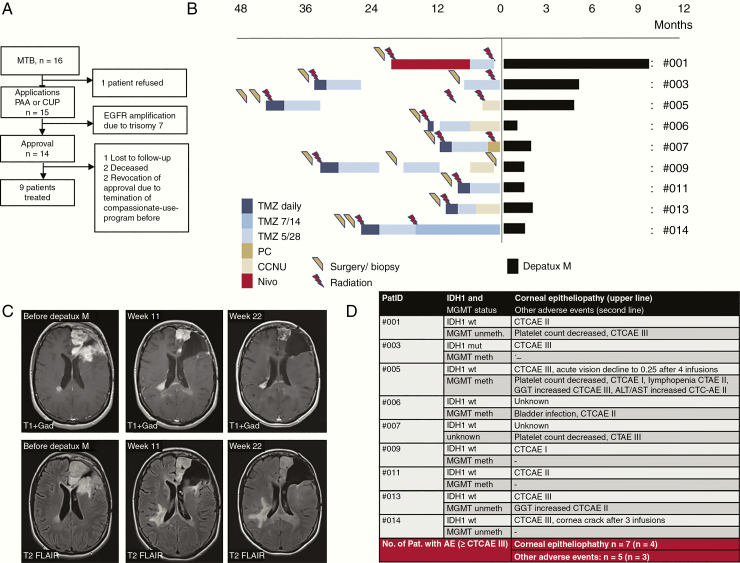
(A) Flow diagram. Sixteen patients were identified in the Molecular Tumor Board. We applied the inclusion into PAA or CUP for 15 patients and received 14 approvals. Eventually, we started Depatux-M treatment in 9 patients. (B) Schematic overview of pretreatments and duration of Depatux-M treatment. CCNU, Lomustine/CCNU chemotherapy; Nivo, nivolumab; PC, procarbazine plus lomustine/CCNU chemotherapy; TMZ, temozolomide. (C) MRI from patient 3 with a partial response at indicated time points after Depatux-M/TMZ initiated. Upper row, gadolinium-enhanced images; lower row, FLAIR images. (D) The table showing adverse events under Depatux-M plus temozolomide treatment. AE, adverse advent; CTCAE, Common Terminology Criteria for Adverse Events. (D) Overview of IDH1 status, MGMT status, and adverse events.

The patients of our cohort had been treated at the University Hospital Tübingen between April 2018 and May 2019 within registered preapproval access and a compassionate use program. The ethics committee approved this analysis. Within the Molecular Tumor Board, we initially identified 16 patients with advanced high-grade glioma and focal EGFR amplification based on molecular profile evaluations (Figure 1A). We eventually started Depatux-M treatment in 9 patients with advanced high-grade gliomas (7 isocitrate dehydrogenase [IDH]1-wildtype glioblastoma, 1 IDH1-mutated glioblastoma, and 1 IDH1-wildtype anaplastic astrocytoma), median age 60 years, (range 29–71 years), and Karnofsky performance score 90% (*n* = 3), 80% (*n* = 2), 70% (*n* = 2), and 60–50% (*n* = 2).

We treated according to the INTELLANCE 2 clinical trial protocol with Depatux-M (1.0–1.25 mg/kg, every 2 weeks) and temozolomide (150–200 mg/m^2^, 5/28 scheme). Pretreatments of all (Figure 1B) included surgery, irradiation, temozolomide, procarbazine plus lomustine/CCNU or lomustine/CCNU chemotherapy alone, and nivolumab. Thus, we treated patients with rather later disease stages compared with INTELLANCE 2 (*n* = 1 first progression, *n* = 3 second progression, and *n* = 5 third to fourth progression) in this preapproval access and compassionate use program.

The treatment durations were 1 cycle (2 infusions) through 8 cycles (15 infusions) (Figure 1B). The first MRI, 1.5–3 months after Depatux-M initiation, showed a partial response according to RANO criteria in 1 patient, stable disease in 2 patients, and progressive disease in 4 patients (Figure 1C). Two patients had clinical deterioration and were not suitable for further MRI follow-up. The median overall survival after initiation of Depatux-M was 4 months (mean 5.1 months, range 1.7–10.9 months). One patient is alive, and another one was lost to follow-up.

We used eye cool packs during infusions for all patients. Corneal epitheliopathy occurred after 2–3 Depatux-M infusions in 7 of 9 patients (Figure 1D). These patients used eye drops containing glucocorticoids 3 times a day and hyaluronic acid-containing liquids every hour. If ocular toxicity ≥ CTC AE grade II occurred, Depatux-M dose was reduced to 1.0 mg/kg. Patient 14 had a cornea crack after 3 infusions, and we stopped therapy. In patient 5, an acute vision decline to 0.25 occurred after 4 infusions, and we stopped the therapy for 5 weeks.

Of note, the INTELLANCE 1 phase III trial using Depatux-M in EGFR-amplified newly diagnosed glioblastoma was discontinued for futility (NCT02573324). The INTELLANCE 2 phase II trial did not detect any significant activity of Depatux M monotherapy at the first progression of glioblastoma, but the Depatux-M/TMZ combination arm became significant with a two 2-year OS analysis of 19.8% (95% confidence interval [CI]: 12.2, 28.8) and 5.2% (95% CI: 1.7, 11.7) in the control arm.^2^ Our observations demonstrate the feasibility of Depatux-M/TMZ in multiple progressing high-grade gliomas but with rather limited activity. A precise refinement of the molecular profile for patient stratification, for example, the threshold for copy numbers or companion molecular alterations, and identification of molecular mechanisms leading to acquired resistance might be further important steps for optimizing clinical development programs of EGFR-targeting ADCs.
